# Evaluation of the Space GlucoseControl System for Managing Stress-Induced Hyperglycemia in ICU Patients: Efficacy and Safety Assessment

**DOI:** 10.2174/0118715303346311250415111740

**Published:** 2025-04-29

**Authors:** Peng-Cheng Liu, Dan-Dan Fan, Qun-Ying Bao, Xiao-Mei Jiang, Juan Lu

**Affiliations:** 1 Blood Purification Center, The Second Affiliated Hospital of Soochow University, Suzhou, 215004, Jiangsu Province, China;; 2 Department of Pharmacy, The Second Affiliated Hospital of Soochow University, Suzhou, 215004, Jiangsu Province, China;; 3 Intensive Care Unit, The Second Affiliated Hospital of Soochow University, Suzhou, 215004, Jiangsu Province, China

**Keywords:** Blood glucose, glucose management, ICU, space glucoseControl system, stress-induced hyperglycemia, focused patient care

## Abstract

**Background:**

Despite the increasing adoption of the Space GlucoseControl system (SGCs), a novel blood glucose management technique, in critically ill patients, its efficacy and safety warrant further investigation.

**Objective:**

This study aimed to assess the efficacy and safety of SGCs in managing patients with stress-induced hyperglycemia in the intensive care unit.

**Methods:**

A prospective study was conducted involving 22 patients with stress-induced hyperglycemia monitored using the SGCs for 72 hours. Measurements included mean blood glucose, maximum blood glucose, minimum blood glucose, and frequencies of hyperglycemia (> 8.3 mmol/L), hypoglycemia (< 4.4 mmol/L), and target blood glucose (4.4 - 8.3 mmol/L).

**Results:**

Mean blood glucose level was significantly different at 24 hours and 48 hours and 24 hours and 72 hours with SGCs (24h *vs* 48h, *P*=0.0289; 24h *vs* 72h, *P*=0.0252). The times of hyperglycemia (> 8.3 mmol/L) (24h *vs* 48h, *P*=0.0289; 24h *vs* 72h, *P*=0.0216) was significantly reduced at 48 hours and 72 hours compared to 24 hours. The average interval between blood glucose measurements was significantly extended at 48 hours and 72 hours compared to 24 hours (24h *vs* 48h, *P*=0.0037; 24h *vs* 72h, *P*=0.0332). The frequency of target blood glucose (4.4 - 8.3 mmol/L) under SGCs blood glucose management at 48 hours and 72 hours was significantly higher than at 24 hours (24h *vs* 48h, *P*=0.0395; 24h *vs* 72h, *P*=0.0379). There was no significant difference in the times of hypoglycemia (< 4.4 mmol/L) and the highest blood glucose values between 24 hours, 48 hours, and 72 hours with SGCs glucose management (*P*>0.05). These findings indicate that SGCs blood glucose management can promote greater stability in the blood glucose levels of patients and alleviate the workload of medical staff to some extent.

**Conclusion:**

SGCs is effective in stabilizing blood glucose levels, increasing the frequency of target blood glucose values, and thereby reducing significant glucose fluctuations, which can improve patient recovery outcomes. Additionally, prolonged use of the SGCs not only optimizes glucose management but also significantly alleviates the workload of nursing staff, enhancing efficiency and allowing for more focused patient care.

## 1 INTRODUCTION

Blood glucose metabolism disorder is a common complication in patients who are in the intensive care unit (ICU) and can be caused by various stressors or injuries. Stress-induced hyperglycemia refers to a temporary increase in blood sugar in patients caused by various stress factors, such as severe trauma, infection, shock, and surgery, including patients with or without a history of diabetes [1, 2]. Normally, the blood sugar level will gradually return to normal as the patient's condition stabilizes [2]. Although there is currently no unified diagnostic criteria for stress-induced hyperglycemia, according to the 2023 Guidelines for the Management of Hospitalization for People with Diabetes published by the American Diabetes Association, stress-induced hyperglycemia can be diagnosed in patients with no history of diabetes whose random blood glucose is greater than 7.8 mmol/L during hospitalization or patients with a history of diabetes whose random blood glucose is greater than 13.9 mmol/L [3]. Stress-induced hyperglycemia is a critical illness indicator in ICU patients and is directly associated with poor prognosis. Studies demonstrated that the mortality rate for patients with stress-induced hyperglycemia is 18.3 times higher than those with normal blood glucose levels and 2.7 times higher than for diabetic patients [4]. Additionally, stress-induced hyperglycemia increases the risk of renal failure, acute myocardial infarction, and cerebrovascular events, and it may also trigger the onset of type 2 diabetes in patients without a prior diagnosis [5, 6]. High-stress states, characterized by increased energy and oxygen consumption as well as heightened catabolic metabolism, can exacerbate tissue and organ dysfunction. On the other hand, hypoglycemia can cause irreversible damage to the brain and nervous system [7]. Therefore, managing blood glucose levels in ICU patients is essential. In recent years, researchers have been investigating more effective blood glucose management strategies for ICU patients [8-10]. Current methods for blood glucose monitoring include peripheral blood glucose testing, Continuous Glucose Monitoring (CGM), Flash Glucose Monitoring (FGM), and the Space GlucoseControl System (SGCs) [11, 12]. While peripheral blood glucose monitoring is widely used, it is time-consuming and labor-intensive. CGM and FGM are more advanced techniques, but their application in ICU patients is limited by factors, such as edema and metabolic disturbances. Additionally, these methods do not sufficiently account for the nutritional needs of ICU patients, making it difficult to accurately determine insulin dosage. SGCs, a newer technology for precise blood glucose control, administer insulin based on factors, such as enteral and parenteral nutrition, weight, and other patient-specific variables. Therefore, SGCs has a clear advantage for critically ill patients who receive nutritional support and need to pump insulin to control their blood sugar. It has been increasingly adopted abroad, with studies showing effective hyperglycemia control and stability [4-7, 13]. However, research on this technique in China is limited, and there is a lack of research on ICU patients with stress hyperglycemia. Hence, this study implemented the SGCs in ICU patients suffering from stress-induced hyperglycemia and observed its effects on blood glucose control over time. The aim was to provide clinical insights for enhancing the quality of blood glucose management. It is expected to provide help for blood glucose management in enteral nutrition patients in ICU in China to promote the early recovery of patients.

## 2 METHODS

### 2.1 Design and Patients

This study utilized a prospective design and conducted objective sampling of the subjects from January, 2019 to October, 2020. A total of 22 patients with stress-induced hyperglycemia were included, and blood glucose management was performed for 72 hours using SGCs. Inclusion criteria were patients with stress-induced hyperglycemia with at least one blood glucose reading ≥ 10.0 mmol/L requiring intravenous insulin pumping, age ≥ 18, and an estimated ICU stay of at least 72 hours. Exclusion criteria were pregnant women, insulin allergies, and patients who were dying or likely to die within 24 hours.

### 2.2 Sample Size Calculations

G*Power 3.1.9.7 was used to calculate the sample size, employing the repeated measures ANOVA within-subjects design method. Based on prior analysis, the significance level (α) was set to 0.05, the statistical power (1-β) to 0.8, and the effect size to 0.35. The repeated measures ANOVA was performed twice according to the study design. For the single-group longitudinal study, the lower limit for spherical correction was set to 1/ (number of repeated measurements - 1) = 1. The calculated sample size was 19 participants, with an additional 10% accounting for potential sample loss, bringing the final estimated sample size to approximately 21 participants.

### 2.3 SGC System and Routine Glucose Management

The SGC system includes (1) two infusion pumps for delivering nutrition, either through the digestive system (enteral) or directly into the bloodstream (parenteral), (2) a push pump to control insulin delivery, and (3) a blood glucose module that features a built-in physiological model of glucose and insulin that simulates patient physiology [8]. By considering factors, such as insulin dosage and enteral and parenteral glucose, the system determines the appropriate insulin infusion rate to achieve optimal insulin dosing. This helps in reducing episodes of hyperglycemia, hypoglycemia, and abnormal blood sugar fluctuations. Furthermore, the module incorporates an algorithm-driven system that predicts the next blood glucose measurement time, calculates potential blood glucose concentrations for the upcoming hour, and adjusts insulin delivery accordingly to maintain stable target blood glucose levels. The algorithm adapts insulin dosage based on observed input-output relationships during blood glucose control, including insulin sensitivity, nutritional changes, drug impact on blood glucose, and patient physiological conditions.

The device is operated by a bedside nurse following the physician's instructions. The SGCs blood glucose management protocol involves several steps [4]: First, the peripheral blood glucose of patients is measured. Next, this value is entered into the blood glucose module. After systematic analysis of the blood glucose of patients, the insulin infusion dose is automatically adjusted and determined. Additionally, the system analyzes the next blood glucose monitoring time and provides reminders through voice-activated alarms.

In this study, the target blood glucose range was defined as 4.4 to 8.3 mmol/L [5]. Blood glucose measurements were conducted using the OptiumXceed glucose meter. A standard 50ml syringe was employed, containing 40 units of insulin diluted in 39ml of 0.9% normal saline for blood glucose management. Procedures for treating hypoglycemia are outlined in Fig. (**1**).

### 2.4 Setting

This study took place in an 18-bed ICU.

### 2.5 Data Collection

Trained nursing staff collected data, including age, gender, BMI, history of diabetes, diagnosis, use of sedatives, diuretics, vasopressors, and steroids.

### 2.6 Outcome Measures

The outcome measures monitored in this study comprised mean blood glucose, maximum blood glucose, minimum blood glucose, frequency of hyperglycemia (> 8.3 mmol/L), frequency of hypoglycemia (< 4.4 mmol/L), frequency of target blood glucose (4.4 - 8.3 mmol/L), and the average interval between blood glucose measurements and insulin administration.

### 2.7 Data Analysis

SPSS 23.0 was utilized to perform statistical analysis on the collected data, employing descriptive statistics for general data. Measurement data following a normal distribution and with homogeneous variance were expressed as mean ± standard deviation. Data not meeting normality or with heterogeneous variance were expressed as quartiles. Given the small sample size, the Friedman test was employed for repeated comparisons across different time points. Adobe Photoshop 18.0 was used for image processing. One-way ANOVA was selected for repeated measurement analysis, and the Tukey method was selected for data processing with a 95% confidence interval.

## 3 RESULTS

### 3.1 General Information

In this study, 22 patients in ICU with hyperglycemia received SGC treatment between January 1^st^, 2019, and March 31^st^, 2022. The mean (SD) age of all patients was 67.6 (13.4) years; 17 (77.3%) were males, 5 (22.7%) were females, and 14 (63.6%) had no history of diabetes. The average BMI (kg/m2) was 24.14±4.45. There were 5 patients with multiple injuries (22.73%), 4 patients with digestive system diseases (18.18%), 7 patients with respiratory system (31.82%), 3 patients with nervous system diseases (13.64%), and 3 patients with circulatory system diseases (13.64%). Thirteen cases (59.09%) used sedatives, 14 cases (63.64%) used diuretics, 8 cases (36.36%) used vasopressors, and 5 cases (22.73%) used steroids (Table **1**).

### 3.2 Evaluation of the Effects of Blood Glucose Management in SGCs

**Fig. (2):**
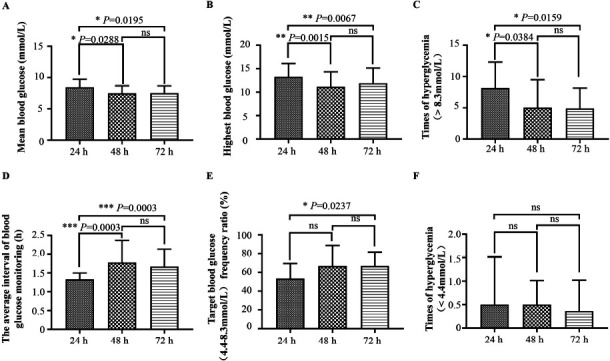
(**A**) Comparing mean blood glucose levels at three 24-hour intervals; (**B**) Comparing peak blood glucose levels at three 24-hour intervals; (**C**) Comparing occurrences of hyperglycemia (> 8.3 mmol/L) at three 24-hour intervals; (**D**) Comparing average intervals between blood glucose monitoring at three 24-hour intervals; (**E**) Comparing frequency ratios of target blood glucose (4.4 - 8.3 mmol/L) at three 24-hour intervals; (**F**) Comparing occurrences of hypoglycemia (< 4.4 mmol/L) at three 24-hour intervals.

The mean blood glucose levels showed significant differences at 24 hours, 48 hours, and 72 hours with SGCs (χ^2^=6.920, *P*=0.031). As presented in (Table **2** and Fig. **2A**), mean blood glucose was significantly different at 24 hours and 48 hours and 24 hours and 72 hours with SGCs (24h *vs* 48h, *P*=0.0289; 24h *vs* 72h, *P*=0.0252). Additionally, the times of hyperglycemia (> 8.3 mmol/L) (24h *vs* 48h, *P*=0.0289; 24h *vs* 72h, *P*=0.0216) was significantly reduced at 48 hours and 72 hours compared to 24 hours (Fig. **2B**), while the mean interval between blood glucose measurements was significantly extended at 48 hours and 72 hours compared to 24 hours (24h *vs* 48h, *P*=0.0037; 24h *vs* 72h, *P*=0.0332) (Fig. **2D**). Moreover, as shown in Fig. (**2E**), the frequency of target blood glucose (4.4 - 8.3 mmol/L) under SGCs blood glucose management at 48 hours and 72 hours was significantly higher than at 24 hours (24h *vs* 48h, *P*=0.0395; 24h *vs* 72h, *P*=0.0237). However, there was no significant difference in the times of hypoglycemia (< 4.4 mmol/L) and the highest blood glucose values between 24 hours, 48 hours, and 72 hours of SGCs glucose management (P>0.05) (Fig. **2C**-**F**). These findings indicate that SGCs blood glucose management can promote greater stability in the blood glucose levels of patients and alleviate the workload of medical staff to some extent.

## 4 DISCUSSION

Two multicenter randomized controlled trials conducted in Europe in 2012 and 2014 established the efficacy of SGCs in blood glucose management [5, 14]. Similarly, two studies conducted in China demonstrated that SGCs significantly better than conventional blood glucose management in controlling blood glucose levels [6, 15].

In this prospective study, we investigated the sustained effects of SGCs on ICU patients with stress-induced hyperglycemia. Our analysis revealed that patients using SGCs equipment experienced gradual stabilization of mean blood glucose levels over time, a significant reduction in the frequency of hyperglycemic episodes, and a steady increase in the proportion of blood glucose levels within the target range. Importantly, there was no increase in the incidence of hypoglycemia. These findings strongly support the clinical efficacy of SGCs in managing stress-induced hyperglycemia in ICU patients, which was in line with previous studies conducted by Amrein *et al*. [4-6, 13, 14]. Furthermore, this longitudinal study is the first to demonstrate the clinical efficacy of SGC in China.

A recent study [16] has demonstrated that an early-stage enteral nutrition program based on the optimal intervention of blood glucose significantly reduces blood glucose fluctuations and improves the quality of blood glucose management. However, this study lacked adequate means for blood glucose monitoring, which increases the workload for medical personnel. The use of SGCs for information-driven blood glucose monitoring in our study addresses this limitation, enabling more scientific management of stress hyperglycemia in patients receiving endo-enteral nutrition support. Furthermore, CGM and intermittent scanning CGM systems offer continuous blood glucose monitoring and provide convenient and comprehensive data to enhance blood glucose management [11]. However, their application in critically ill patients, especially those receiving enteral and parenteral nutrition support, cannot predict the impact of enteral or parenteral nutrition on blood glucose. On the other hand, SGCs can more accurately measure the amount of insulin that a patient needs to pump according to the speed and volume of nutrient solution infusion. This prediction helps to more accurately control the blood glucose of the patient, avoid large fluctuations in blood glucose, further improve the quality of blood glucose management, and promote the recovery of the patient. Therefore, SGCs are recommended for blood glucose management in ICU patients with stress hyperglycemia accompanied by enteral or parenteral nutrition.

Furthermore, SGCs-based blood glucose management enables more precise monitoring of fluctuations due to nutrition or other treatments at any given time. It adjusts insulin dosages to maintain stable blood glucose levels, thereby mitigating the impact of hyperglycemia and hypoglycemia on patient treatment and recovery. These findings are consistent with clinical studies conducted by Amrein *et al*. [4-7, 13, 14]. Additionally, regarding the average interval between blood glucose measurements, initial monitoring frequencies were higher, which increased the nursing staff's workload. However, over time, the monitoring intervals gradually lengthened, reducing the frequency of measurements and thereby easing the nursing workload to some extent. This approach enhances patient outcomes, particularly for general ICU patients with unstable blood glucose, who initially required frequent monitoring (*e.g.*, every 1-2 hours) to achieve stable blood glucose levels with insulin assistance. As SGCs are applied to manage stress-induced hyperglycemia in the ICU, the monitoring intervals lengthen over time, reducing fingertip pricks, which decreases patient discomfort and lowers the risk of fingertip infections. Moreover, stabilized blood glucose levels contribute to overall patient stability and assist in disease management and recovery. This could potentially reduce hospital stays and healthcare costs associated with stress-induced hyperglycemia in ICU patients while also alleviating the physical and emotional burdens on patients’ families. These benefits merit further investigation in future studies.

This longitudinal study highlights the advantages of SGCs and, notably, represents the first investigation in China to assess its safety and efficacy through long-term analysis, demonstrating its effectiveness in managing long-term glycemic control for ICU patients. However, there are several limitations. First, the study did not employ a randomized controlled trial design, which limits the strength of the evidence and may affect the reliability of the data to a certain extent. Second, although the sample size is sufficient for assessing the significant impact of SGCs on managing blood glucose in patients with severe hyperglycemia receiving nutritional support, it remains relatively small. Moreover, it may be difficult to reveal the underlying patterns in this study, affecting the reliability and reproducibility of the results. Future research should involve multi-center studies with larger sample sizes. Additionally, the study population had a diverse range of underlying conditions, suggesting the need for a multi-population classification study to evaluate how different diseases and treatments affect blood glucose levels.

As this is a before-and-after study with an inherent self-control design, potential confounders were minimized. The intervention was applied within the same group of participants, and we did not have a separate control group. Therefore, baseline variability between groups, which often serves as a confounder, is less of a concern here. However, we acknowledge that factors, such as the composition of the nutritional solution, could influence the results. Since the components were automatically standardized by the system, their potential impact is minimized and unlikely to significantly affect the overall findings. Additionally, the differences in underlying conditions between patients had a low impact on the study’s results, further supporting the robustness of the findings.

In this study, the target blood glucose range was determined based on the SGC blood glucose module's range of 4.4 - 8.3 mmol/L. However, reports by Xu *et al*. and Qian *et al*. suggest target ranges of 5.8 - 8.9 mmol/L or 8 - 10 mmol/L for SGCs [6, 17]. Further research is needed to standardize the optimal target blood glucose range for effective management.

## CONCLUSION

SGCs effectively manage blood sugar levels in ICU patients with stress-induced hyperglycemia under enteral or parenteral nutrition support, enabling rapid reduction of blood glucose to target ranges and minimizing variability, thus maintaining stable blood sugar levels. Over time, SGCs reduce the incidence of hyperglycemia without increasing the risk of hypoglycemia. Overall, SGCs demonstrate a favorable effect on blood glucose control. Particularly beneficial during nutrition support, SGCs sustain long-term blood glucose stability and mitigate significant glycemic fluctuations, thereby promoting patient recovery. Additionally, SGCs can extend the time it takes to detect blood sugar as time progresses, ultimately reducing the workload for nursing staff. Therefore, this study recommends that SGCs be used as a priority for glycemic management in patients with stress hyperglycemia who require enteral nutrition or parenteral nutrition support.

## Figures and Tables

**Fig. (1):**
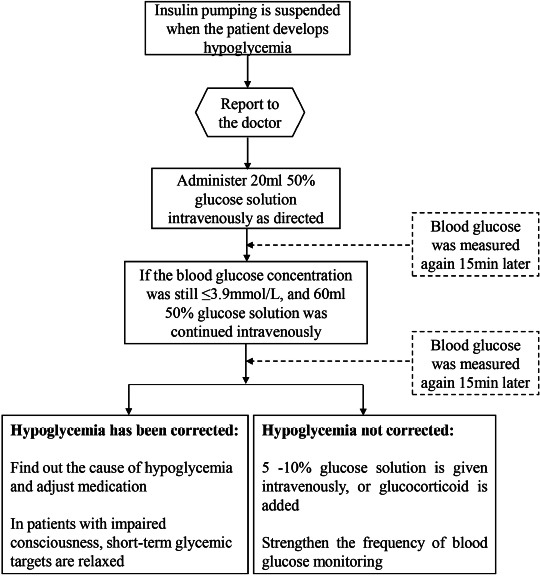
Procedures for dealing with hypoglycemia.

**Table 1:** Line General information for patients with SGCs blood glucose management (N=22).

Characteristic	x̄±s	Cases(n)	Assessing the Constituent Ratios (%)
Age	67.64±13.43	-	-
Gender	-	-	-
Male	-	17	77.27
Female	-	5	22.73
BMI (kg/m^2^)	24.14±4.45	-	-
History of diabetes	-	-	-
Have diabetes	-	8	36.36
Diabetes-free	-	14	63.64
Disease diagnosis	-	-	-
Multiple injuries	-	5	22.73
Digestive system	-	4	18.18
Respiratory system	-	7	31.82
Nervous system	-	3	13.64
Circulatory system	-	3	13.64
Whether to use sedatives	-	-	-
Yes	-	13	59.09
No	-	9	40.91
Whether to use diuretics	-	-	-
Yes	-	14	63.64
No	-	8	36.36
Whether to use vasopressors	-	-	-
Yes	-	8	36.36
No	-	14	63.64
Whether to use steroids	-	-	-
Yes	-	5	22.73
No	-	17	77.27

**Table 2:** Blood glucose management in SGCs at different time points: 95%.

Item	24h *vs* 48h	24h *vs* 72h	48h *vs* 72h
Mean blood glucose (mmol/L)	(0.10, 1.84)	(0.08, 1.82)	(-0.89, 0.85)
Highest blood glucose (mmol/L)	(-0.11, 4.36)	(-0.85, 3.62)	(-2.98, 1.50)
Times of hyperglycemia (Freq)	(0.27, 6.01)	(0.40, 6.14)	(-2.73, 3.01)
Times of hypoglycemia (Freq)	(-0.55, 0.55)	(-0.41, 0.68)	(-0.41, 0.68)
Target blood glucose (4.4-8.3mmol/L) frequency ratio (%)	(-26.02, -0.52)	(-26.12, -0.61)	(-12.84, 12.66)
Average interval of blood glucose monitoring (h)	(-0.77, -0.13)	(-0.66, -0.02)	(-0.21, 0.43)

## Data Availability

All data generated or analyzed during this study are included in this published article.

## LIST OF ABBREVIATIONS

SGCs: Space GlucoseControl System
ICU: Intensive Care Unit
CGM: Continuous Glucose Monitoring
FGM: Flash Glucose Monitoring

## STANDARDS OF REPORTING

STROBE guidelines were followed.
